# Moderate treadmill running exercise prior to tendon injury enhances wound healing in aging rats

**DOI:** 10.18632/oncotarget.7381

**Published:** 2016-02-14

**Authors:** Jianying Zhang, Ting Yuan, James H-C. Wang

**Affiliations:** ^1^ MechanoBiology Laboratory, Departments of Orthopaedic Surgery and Bioengineering, Mechanical Engineering and Materials Science, and Physical Medicine and Rehabilitation, University of Pittsburgh, Pittsburgh, PA, USA; ^2^ Department of Orthopaedic Surgery, Shanghai Sixth People's Hospital Affiliated to Shanghai, Jiaotong University, Shanghai, China

**Keywords:** aging rat, tendon stem cell, treadmill running, proliferation, wound healing, Gerotarget

## Abstract

The effect of exercise on wound healing in aging tendon was tested using a rat moderate treadmill running (MTR) model. The rats were divided into an MTR group that ran on a treadmill for 4 weeks and a control group that remained in cages. After MTR, a window defect was created in the patellar tendons of all rats and wound healing was analyzed. We found that MTR accelerated wound healing by promoting quicker closure of wounds, improving the organization of collagen fibers, and decreasing senescent cells in the wounded tendons when compared to the cage control. MTR also lowered vascularization, increased the numbers of tendon stem/progenitor cells (TSCs) and TSC proliferation than the control. Besides, MTR significantly increased the expression of stem cell markers, OCT-4 and Nanog, and tenocyte genes, Collagen I, Collagen III and tenomodulin, and down-regulated PPAR-γ, Collagen II and Runx-2 (non-tenocyte genes). These findings indicated that moderate exercise enhances healing of injuries in aging tendons through TSC based mechanisms, through which exercise regulates beneficial effects in tendons. This study reveals that appropriate exercise may be used in clinics to enhance tendon healing in aging patients.

## INTRODUCTION

Aging is known to adversely affect the human body and lead to degenerative changes in tissues and organs [1]. Specifically, aging predisposes tendons to develop tendinopathy, and causes tendons to frequently rupture and re-rupture [2]. Aging rats (22 months) also have decreased activity and increased risk for tendon injuries; they exhibit decreased amounts of Collagen-I, -III and -V, and proteoglycan 4 (PRG4) in the Achilles and tibialis anterior tendons [2]. Furthermore, aging increases the nucleus to cytoplasm ratio in tendons, increases lipid deposition, and decreases vascularization. Additionally, aging lessens the number of tendon cells, decreases their activity [3], reduces the integrity of tendon matrices [4] and the response of tendon cells to cellular stimuli [3]. A recent study showed that aging affects the responses of human Achilles and patellar tendons to transverse strain (TS) by reducing the TS ∼2.5% every 10 years of life [4]. Consequently, the mechanical strength of aging tendons decreases making it susceptible to injuries, which lowers the quality of life among the millions of aging population [5].

Contrary to aging, mechanical loading in the form of exercise is well known to exert beneficial effects on tendons [6]. In general, tendons adapt to mechanical loads, which also influence tendons' mechanical properties [7] [8]. For example, an exercise regimen performed for 14 weeks by young recreational runners increased the maximum plantar flexion muscle strength and triceps surae [9], tendon-aponeurosis stiffness and increased the running economy [10]. Similarly, increased patellar tendon microcirculation and reduced tendon stiffness was observed in athletes after performing knee extension eccentric exercises [11]. These changes are induced by the mechanical load-induced external strain, which is transmitted to the tendon cells *via* the tendon matrix and subsequently changes the cellular responses of tendon cells based on the type of mechanical load experienced [6, 12]. For instance, moderate mechanical loading such as a moderate treadmill running (MTR) regimen enhances anabolic changes in mice Achilles and patellar tendons and yet large mechanical loading such as an intensive treadmill running (ITR) promotes catabolic changes [6, 13]. Such benefits of moderate exercise have been well established in both human and animal models by a number of studies [14-21].

However, the effect of exercise on wound healing in aging tendons is largely unknown. In recent studies, we demonstrated the critical role of tendon stem/progenitor cells (TSCs) in regulating the beneficial or detrimental effects of tendons in animal models [13, 22-24]. Like adult stem cells, TSCs are essential for the maintenance and repair of tendinous tissues when injured. In this study, we tested the hypothesis that healing of a tendon injury is improved in aging rats that performed MTR exercise prior to the injury and that these effects are likely achieved through TSC-based mechanisms.

## RESULTS

### Effect of MTR on intact aging tendons

**Figure 1:**
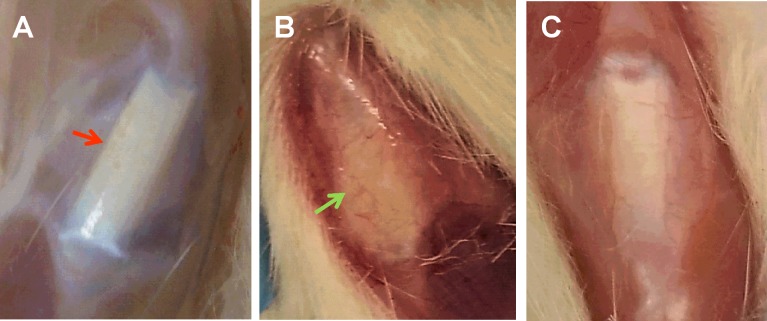
The effect of MTR on gross morphology of aging rat patellar tendons **A.** Young tendon (3 months); **B.** Aging tendon (20 months); **C.** Aging tendon after MTR. Compared to young tendon **A.** aging tendon **B.** appears vascular and less intact. After MTR **C.**, aging tendon has regained its intact structure and has decreased number of blood vessels.

**Figure 2:**
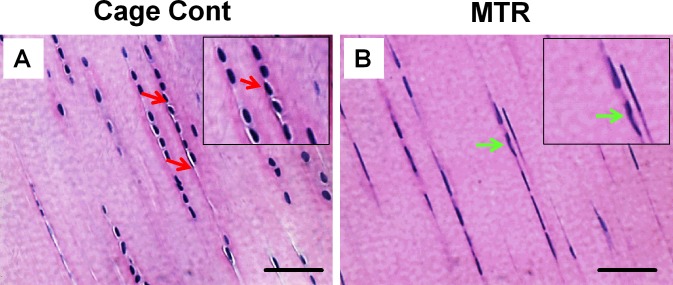
The effect of MTR on the structure and cellularity of aging rat patellar tendon **A.** Tendon section from cage control aging rat. **B.** Tendon section from aging rat after MTR. It is evident that MTR increases the number of typical tendon cells that are more elongate (green arrows) **B.** and decreases the number of round shaped cells presumably non-tenocytes (red arrows) **A.**. Insets represent magnified sections. Bars: 50 μm.

Morphological observations revealed intact, glistening, white and healthy patellar tendons in young rats (Figure 1A, 3 months old). In contrast, tendons in the control aging rats (20 months old) appeared yellowish and highly vascular (Figure 1B, green arrow). Particularly, these aging tendons did not have a defined shape observed in the young tendon. However, tendons in aging rats after MTR regimen prior to wounding were not as yellowish, had largely regained their intact nature and the blood vessels over the tendon were also reduced (Figure 1C). Moreover, H&E staining showed the presence of more round shaped cells (Figure 2A, red arrows) in the cage control group, while in the MTR group more cells were elongated (Figure 2B, green arrows), which is the typical shape of TSCs observed in young rats [25].

### MTR improves tendon wound healing in aging rats

**Figure 3:**
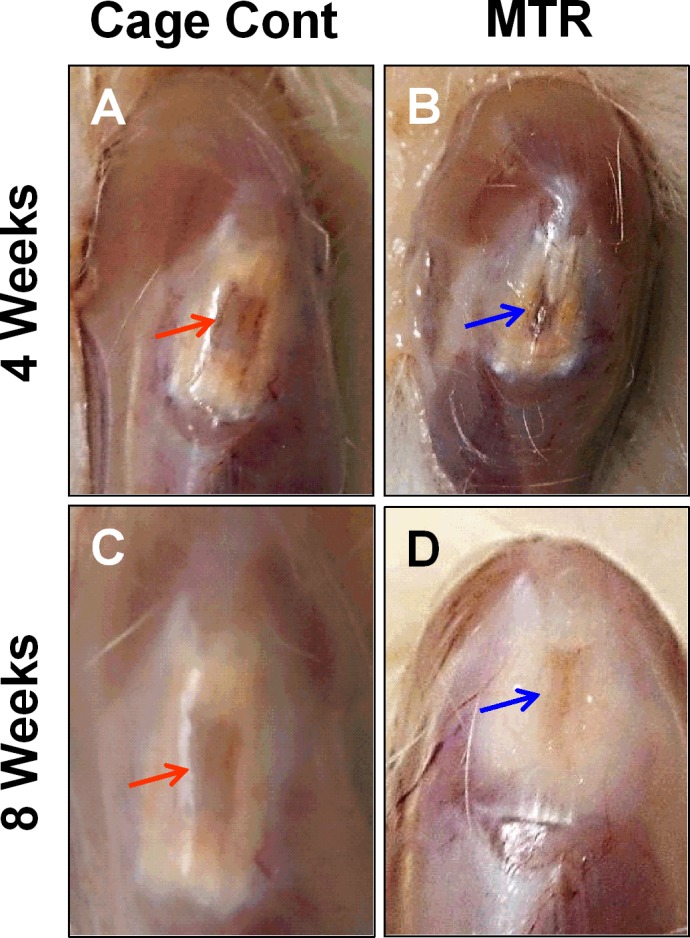
The effect of MTR on wound healing in aging rat patellar tendon **A., C.** Control aging tendons. **B., D.** Tendons from aging rats after MTR. At 4 weeks, the window defect in control aging rats **A.** appears bruised and vascular. But MTR accelerated healing of the wound **B.**, which is evidently smaller and seems to be closing (blue arrow). At 8 weeks, the wound in the control group still looks bruised and vascular (red arrow) **C.**, whereas in the MTR group **D.** the wound for the most part appears to be healed.

**Figure 4:**
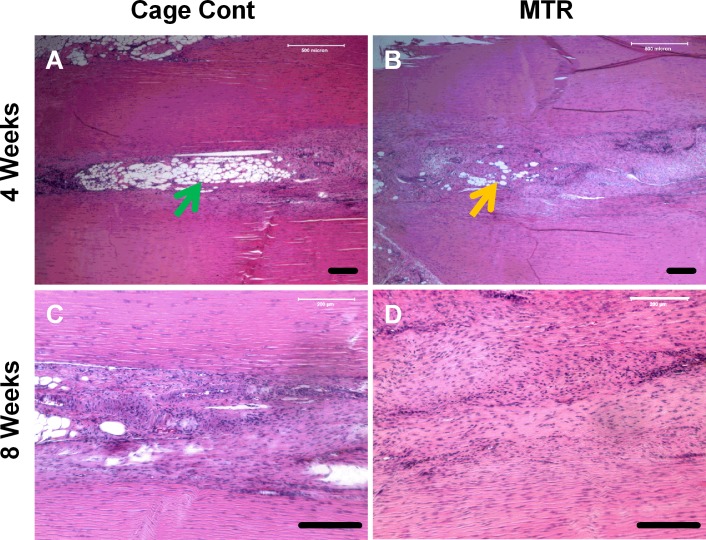
Histological analysis of wound healing in aging rat patellar tendons **A., C.** Control aging tendon; **B., D.** Aging tendon from rats after MTR. At 4 weeks, the control aging tendons have large numbers of lipid-like structures in the wound site (green arrow) **A.**. But these structures are reduced in aging tendons after the MTR regimen (yellow arrow) **B.**. At 8 weeks, the control aging tendon appears to be healing but the lipid-like structures still remain **C.** while in the MTR group, the normal-like tendon structure is regained in aging tendons, which have well organized matrix and evenly distributed cells **D.**. Bars: 200 μm.

The effects of pre-exercise on tendon wound healing were determined by allowing aging rats to run on the MTR regimen and then wounding the patellar tendons after the run. Wounds in the cage control aging rats without mechanical loading appeared to have a large unhealed area and visible blood vessels 4 weeks after wounding (Figure 3A, red arrow). In the MTR group, a similar wound in the rat patellar tendons had healed significantly; this wounded region had poor vascularization and only a small unhealed area was visible. Besides, tendon tissue appeared to be closing in on the unhealed area (Figure 3B, blue arrow). After 8 weeks, healing of the tendon wound in the cage control had largely improved but the unhealed portion was apparent and appeared immature (Figure 3C, red arrow). In contrast, wound healing in the MTR group was almost complete with the wound almost invisible, reduced redness and vascularity, and visibly intact tendon beneath the skin (Figure 3D, blue arrow). A major morphological difference between the control and MTR groups was that the tendon wound in the control group showed a pinkish ‘tendon-less’ area while the same area had thickened in the MTR group indicating new tendon formation (Figure 3C, 3D). This observation was supported by H&E staining; after 4 weeks the control tendon sections had large numbers of lipid-like structures particularly in the middle of the tendon wound (Figure 4A), which corresponds to the unhealed tendon wound in morphological observations (Figure 3A). However, in the MTR group, the tendon sections had lesser numbers of lipid-like structures (Figure 4B). After 8 weeks, tendon wound healing was more advanced in the MTR group (Figure 4D) with uniform cell distribution and horizontal cell organization unlike in the control group with unevenly arranged cells and several lipid-like structures (Figure 4C). These lipid-like structures were completely absent in the MTR group (Figure 4D). These results indicate that pre-exercise in the form of MTR can accelerate tendon wound healing in aging rats.

### MTR decreases the presence of senescent tendon cells in aging rats

**Figure 5:**
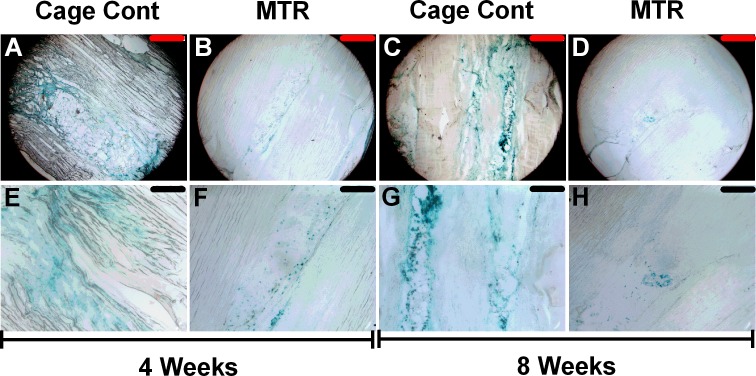
β-gal staining of healing wound in aging rat patellar tendon At 4 weeks, over 90% of wound area in the control aging tendons stained positive for the senescence marker, β-gal **A.**-**E.**; however, less than 30% of wound area in MTR group was positive for β-gal **B.**-**F.**. At 8 weeks, senescent marker expressing cells were still found in control aging tendons **C.**-**G.**, while only a few cells in MTR group were positive for β-gal **D.**-**H.**. Red bars: 500 μm; Black bars: 200 μm.

To further evaluate the effects of mechanical loading on aging tendon tissue, we investigated the effects of MTR on senescent cells in the patellar tendons of aging rats. Our results showed that after 4 weeks, over 90% of the wound area in the cage control tendons stained positive for the senescence marker, β-gal (turquoise blue, Figure 5A-5E); however, less than 30% of the tendon wound in the MTR group stained positive for β-gal (Figure 5B-5F). Similar but more pronounced decrease was observed after 8 weeks in MTR group (Figure 5D-5H) while the decrease in the control group was not marked (Figure 5C-5G). At a higher magnification, large gaps are also visible in the cage control group (Figure 5C-5G) and may be caused due to the weak tendon structure collapsing during the sectioning of tendon tissue. In contrast, the MTR group does not have any gaps (Figure 5D-5H) likely due to the increased strength and structure of these tendons that hold together during the sectioning process.

### MTR increases proliferation of aging TSCs

**Figure 6:**
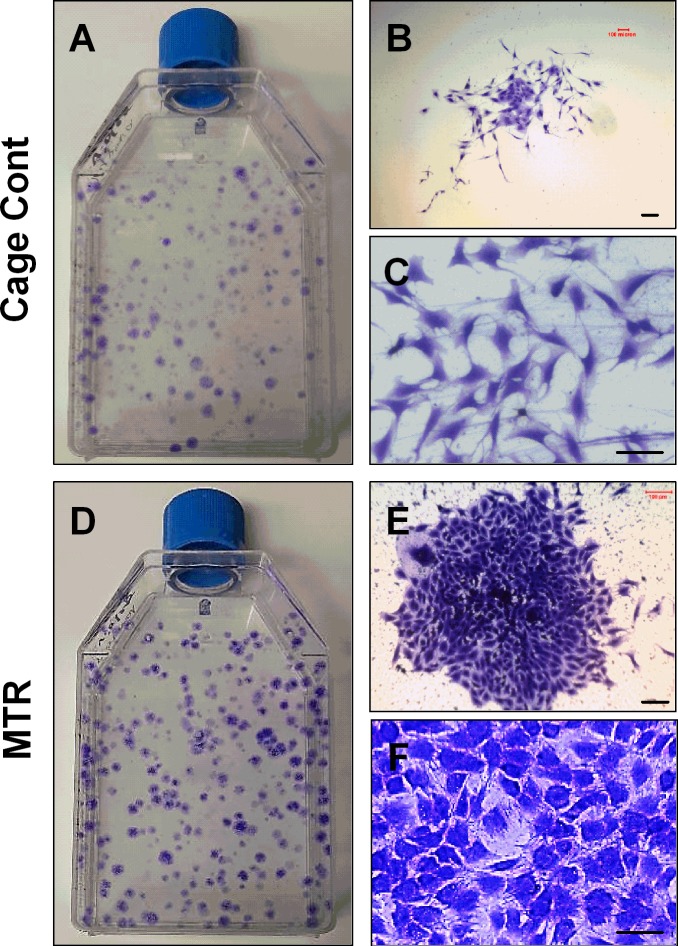
MTR increases the colony number and colony size of TSCs from aging rat patellar tendons **A., B., C.** - TSCs from control aging rats; **D., E., F.** - TSCs from aging rats after MTR. The number of colonies in control aging rats **A.** is much smaller than those in aging rats after MTR regimen **D.**. The number of cells per colony was also lower in the control group **B., C.** than MTR group **E., F.**. Note that the cells in MTR group **F.** are cobblestone shaped, the typical shape of “authentic” TSCs, while many cells in cage control group **C.** are spindle shaped. Bars: 100 μm.

**Figure 7:**
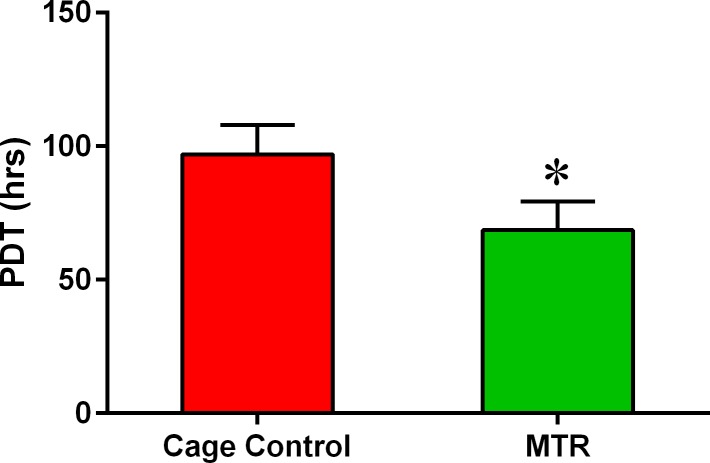
MTR enhances the proliferative potential of aging rat TSCs TSCs were isolated both from MTR and control groups. It is shown that TSCs in MTR group grew much quicker than control group because the former had a reduced PDT compared to control group (*P < 0.05).

Next, we addressed the cellular effects induced by MTR on the wound healing in aging rat patellar tendons by investigating TSCs. When cultured *in vitro*, aging rat TSCs in the cage control and MTR groups differed in three specific aspects; number of TSC colonies, size of each colony and the numbers of cobblestone shaped cells. The number of colonies was evidently higher in the MTR group than the cage control (Figure 6A-6D). Such higher number of TSC colonies in the MTR group is typically observed in TSCs isolated from young rat tendons [6]. Besides, the MTR group had more cells in each colony (Figure 6E) a higher percentage of these cells were cobble-stone shaped (Figure 6F) when compared to the cage control (Figure 6B). Furthermore, TSCs in the MTR group were arranged in a monolayer (Figure 6E, 6F) unlike the cage control where some cells were clumped together (Figure 6B, 6C). These results were also supported by the TSC proliferation data, which showed that population doubling time (PDT) of aging TSCs from rats on an MTR regimen was ∼35% less than the cage control (Figure 7) indicating less time for these TSCs to double in number.

### MTR increases the extent of stem cell marker expression in aging TSCs

**Figure 8:**
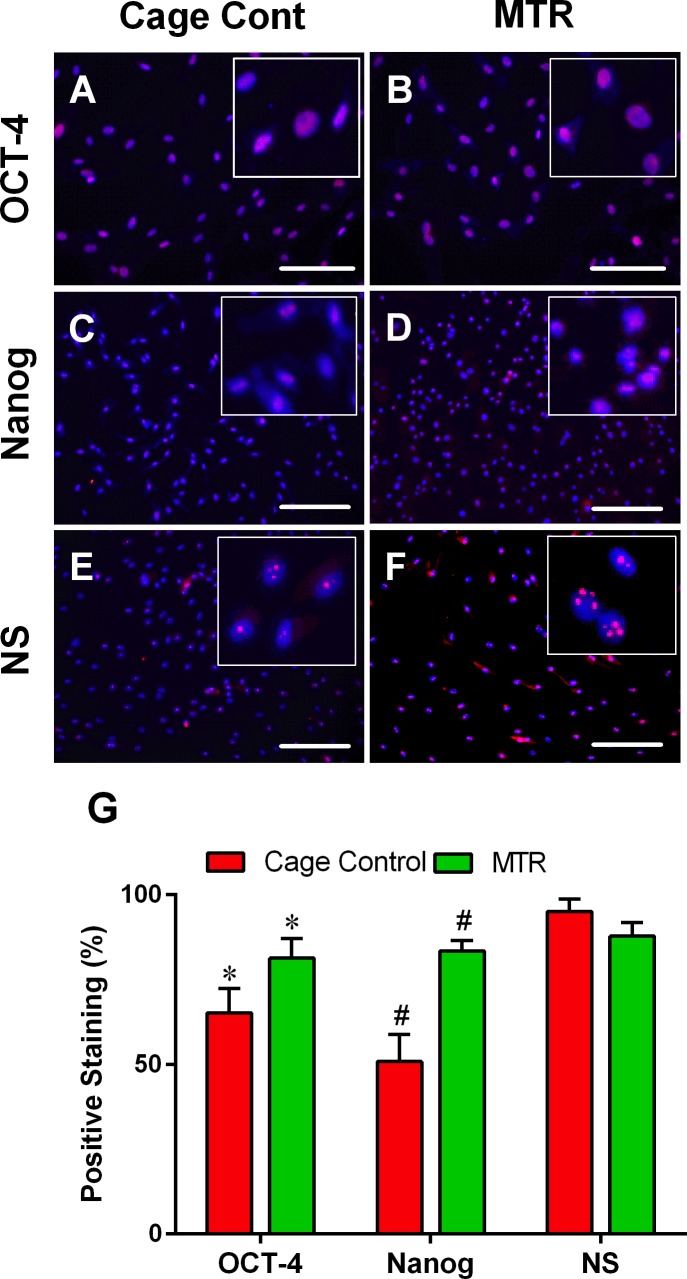
MTR enhances stem cell marker expression in TSCs from aging rats Expression of the three stem cell markers, OCT-4, Nanog and nucleostemin (NS) is minimal in the control aging rat TSCs **A., C., E.** but their expression level is increased in aging rats on an MTR regimen **B., D., F.**. Semi-quantification **G.** of these stem cell markers by counting the positively stained cells showed that MTR increased OCT-4 and Nanog expression but not NS. Bars: 100 μm, *P < 0.05 MTR *vs*. the respective control.

Immunohistochemical staining of the three stem cell markers OCT-4, Nanog and nucleostemin (NS) was minimal in the cage control group (pink dots, Figure 8A, 8C, 8E) but marginally higher in TSCs from the rats in the MTR group (pink dots, Figure 8B, 8D, 8F). These results were in alignment with the semi-quantification data showing significant increases in OCT-4 (20 %) and Nanog (39 %) staining in the MTR group in comparison with the cage control (Figure 8G). However, staining for NS in the TSCs from the MTR group was 8 % lower than the control group (Figure 8G).

### MTR up-regulates tenocyte related genes in aging TSCs

**Figure 9:**
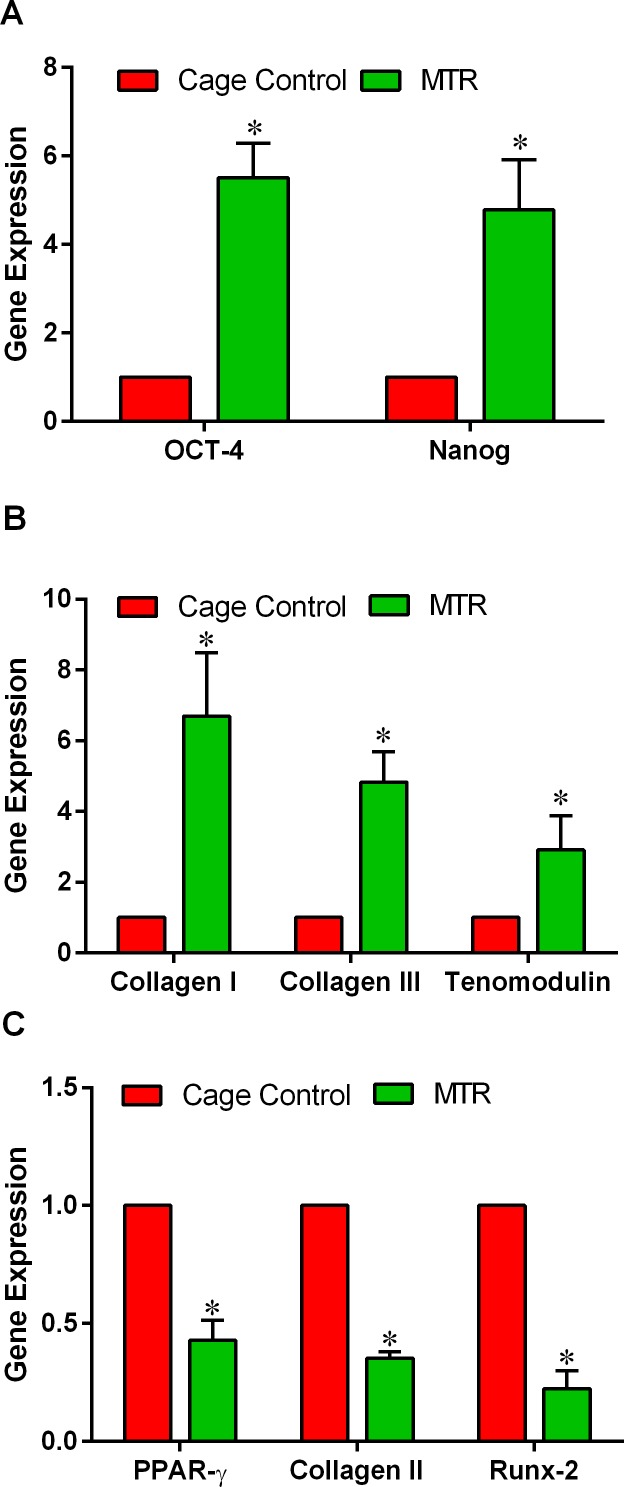
MTR enhance both stem cell and tenocyte related marker expression but decreases non-tenocyte related gene expression in aging rats **A.** Stem cell marker genes, OCT-4 and Nanog; **B.** Tenocyte related genes, Collagen I, Collagen III, and tenomodulin; **C.** Non-tenocyte related genes, PPAR-γ, Collagen II, and Runx-2. Compared to the control, MTR markedly increased the expression of both stem cell marker genes as well as tenocyte related genes. In contrast, all three non-tenocyte related genes decreased > 50% when aging rats were subjected to MTR. *P < 0.05 MTR compared to the respective control.

The gene expression levels of tendon stem cell markers, tenocyte and non-tenocyte markers in the cage control and MTR groups were measured by qRT-PCR. Expression of the stem cell marker genes, OCT-4 and Nanog remained low in the TSCs from the cage controls however, after MTR, the expression of these two genes were 5- and 4-fold higher than the cage control, respectively (Figure 9A). In a similar manner, MTR significantly enhanced the expression of tenocyte related genes including Collagen I (5-fold), Collagen III (3-fold) and tenomodulin (2-fold) more than the cage control TSCs (Figure 9B). However, an opposite trend was observed in the expression of non-tenocyte related genes PPAR-γ, Collagen II and Runx-2; MTR significantly reduced the expression level of these genes by more than 50% (Figure 9C).

## DISCUSSION

Tendons in aging patients are prone to injuries because of reduced mechanical strength due to compromised tendon structure. However, healing of tendon injuries is slow [26-28]. Therefore, in this study, we investigated whether exercise in the form of moderate treadmill running (MTR) prior to an injury can be a preemptive method that augments wound healing in an aging rat (20 months) model. Our results showed that MTR may benefit aging tendons in the following manners: by suppressing tendon cell senescence and accelerating the elimination of senescent tendon cells. These changes are likely mediated by the effects of MTR on TSCs, which had increased anabolic changes and reduced catabolic changes in aging rats. Specifically, MTR increased TSC proliferation, up-regulated tenocyte related genes (Collagen I, Collagen III and tenomodulin) and down-regulated non-tenocyte genes (PPAR-γ, Collagen II and Runx-2). All these processes collectively reduced the number of senescent cells and enhanced collagen fiber organization thus accelerating wound healing in aging rats. These findings support our working hypothesis in this study that moderate exercise augments wound healing in aging rat tendons through TSC-based mechanisms. Thus, our study indicates that routine moderate exercises should be recommended to the aging population as a preemptive protocol to quicken wound healing in the event of tendon injuries in aging individuals.

Aging is known to decrease the functional competence of the body. In tendons, aging causes a number of changes; it increases tendon stiffness, changes TSC morphology from elongated and uniform in young tendons to large, flat and heterogeneous in aging tendons [29], decreases TSC proliferation, down-regulates the expression of stem cell markers (OCT-4, nucleostemin, Sca-1 and SSEA-1), and tenocyte related markers (Collagen I and tenomodulin) [24], reduces collagen synthesis and turnover [30, 31], increases the senescent marker, CCN-1 [32], and causes other related changes. Emerging evidence suggests that these aging related changes can be reversed by the effects of moderate exercise such as MTR on TSCs [6, 22, 24], which play a critical role in tendon maintenance and repair. TSCs execute these roles by their capacity to self-renew and differentiate into tenocytes, which are the predominant cell type in tendons [22], and are responsible for producing extracellular matrix proteins (e.g. collagens and proteoglycans) and remodeling tendon structure during recovery from an injury or degenerative changes [22]. Because TSCs self-renew, they are in a “stand by” mode to replenish lost cells and repair injured matrix when needed. However, as aging progresses TSC proliferation is decreased and their normal ability to differentiate into tenocytes shifts into aberrant non-tenocyte differentiation [24, 33].

Recently, using thickness shear mode (TSM) resonators we showed that aging rat tendon cells exhibit increased stiffness, along with extensively changed TSC morphology (large, flat and heterogeneous) when compared to young TSCs (elongated and uniform) [29]. This indicates that the altered TSC morphology and the likely molecular changes that concurrently occur may contribute to the increased stiffness in aging tendons. In the present study, MTR clearly reversed the deteriorating morphology of aging TSCs *in vitro* from a clumpy to more homogenous cell population (Figure 6). Moreover, MTR also changed tendon cell morphology *in vivo* from rounded to a more elongated form (Figure 2), which is typically observed in young TSCs [25]. In a previous study, MTR also strengthened aging rat tendons (24 months) by inducing anabolic changes such as increasing TSC proliferation, and the expression of OCT-4, nucleostemin, Sca-1 and SSEA-1 (stem cell markers), and Collagen I and tenomodulin (tenocyte related markers) [24], which is consistent with this study. Together, these and our present studies on aging rats clearly suggest that while aging deteriorates TSC function, MTR can mitigate/reverse these age related detrimental changes in tendons by impacting TSCs; that is, MTR can restore aging TSCs to more young TSCs like (Figures 2, 6) by up-regulating stem cell marker expression (Figures 8, 9) and down-regulating non-tenocyte markers (Figure 9).

Another significant effect of aging on tendons is the poor organization of collagen fibers in the aging *versus* the young tendons, which have well-organized collagen fibers. It has been reported that in aging tendons collagen content reduces in an age-dependent manner; that is, collagen synthesis as well as collagen turnover diminish with increasing age [30, 31]. Higher crosslinking of the tropocollagen [34] then ensues and increases the mechanical stiffness of tendons [35, 36] thus reducing the mechanical properties of aging tendons. However, exercise has the opposite effects on collagen synthesis in tendons. Several studies have shown that acute exercise in young humans increased anabolic processes causing a net increase in collagen I in the Achilles tendons [17, 37, 38]. We previously showed that in mice, MTR increased the collagen production/expression; young mice (2.5 months old) on MTR had increased collagen production [22] and aging mice (9 months old) on an MTR regimen had higher Collagen I gene expression [24] in TSCs. This study also revealed a higher gene expression of Collagen 1 (4-fold) and Collagen III (2-fold) in the MTR group (Figure 9). Moreover, H&E staining of the aging tendons also showed more collagen fibrils and their better organization after MTR (Figure 4) thus indicating that MTR's ability to accelerate wound healing in aging tendons may be linked to the increase in the collagen content in the aging tendons.

In many tissues, aging has been known to induce cell senescence [39, 40], which deter wound healing [41]. Our recent study on an animal model found that the expression of CCN-1, a senescent protein, in TSCs increased in an age-depended manner [32]. Similarly, treating aging mouse TSCs with CCN-1 *in vitro* resulted in the following senescence related changes: marked increase in SA-β-Gal accumulation, down-regulation of tenocyte related genes (Collagen I and tenomodulin) and up-regulation of senescence-related inflammatory genes (interleukins IL-1α, IL-1β, IL-6, IL-10rβ, IL-12β, MMP-13 (matrix metalloproteinase-13), and GRO-1 (chemokine (C-X-C) motif ligand-1), in a CCN-1 dose-dependent manner [32]. Our present study showed that these senescent related changes could be mitigated by MTR because MTR decreased β-gal positive cells in aging rat tendons (Figure 5). Thus, moderate exercise can improve aging tendon's cellular structure and mechanical strength by reducing and/or eliminating senescent cells in aging tendons. Besides, the anabolic changes simultaneously induced by MTR also promote TSC proliferation and tenocyte formation that replace the eliminated senescent cells and thereby repopulate aging tendons with “young and able” tendon cells.

Our rationale to use MTR as an exercise model in this study was based on our previous study demonstrating that in young mice MTR induced anabolic effects in the patellar and Achilles tendons by inducing TSCs to proliferate at a higher rate and synthesize more collagen than the control mice that were sedentary [22]. Similarly, MTR also induced up-regulation of tenocyte related genes (Collagen I, tenomodulin) that help form tendon tissues but did not affect non-tenocyte-related genes (LPL, Sox-9, Runx-2, Osterix) [6] that often result in the formation of non-tendon tissues. In contrast, an intensive treadmill running (ITR) protocol produced detrimental effects by up-regulating not only the tenocyte related genes but also the non-tenocyte-related genes [24] that result in the formation of non-tendon tissues such as fatty, bone-like and cartilage-like tissues often found in the tendons of aging individuals and patients with chronic tendinopathy [31]. These non-tendon tissues compromise tendon structure and reduce tendon's biomechanical properties. In contrast, MTR increases the formation of tendon tissues and reduces non-tendon tissues thus likely promoting tendon structure as shown in this study (Figure 4). These findings indicate that only appropriate mechanical loading could benefit tendons because of their potential to specifically induce tendon tissue formation by promoting anabolic changes in TSCs. Excessive mechanical loading may induce differentiation of TSCs into non-tenocytes thus promoting the development of non-tendinous tissue and degenerative tendinopathy in patients.

Based on the findings of this study, we propose that prior to surgical intervention aging patients with tendon injuries should be recommended to follow an MTR regimen that may promote healing of the injury. Our findings reveal that this pre-exercise protocol will fasten the healing process through TSC-based mechanisms when compared to traditional surgeries. This may also be an economic and risk free option to the currently available treatment of tendon injuries using drugs such as non-steroid anti inflammation drugs (NSAIDs). Thus, the findings of this study are relevant not only to prevent injuries and augment wound healing in aging tendons but could also encourage the young population to avoid a sedentary mode of life because load deprivation is known to decrease collagen synthesis and increase collagen turnover [42]. Moreover, moderate exercise not only accelerates wound healing in weight bearing tendons as shown in this study, but also improves the mechanical properties of non-weight bearing tendons such as rat tail tendons through systemic effects [15].

One of the limitations of this study is that we did not investigate the mechanical properties of the aging tendons in rats on the MTR regimen. Future studies should address whether the MTR induced morphological and molecular changes in TSCs may decrease the stiffness of aging tendons. Second, this study does not shed light into the molecular signaling mechanisms responsible for the increase in the proliferation potential of aging TSCs. While a number of molecular components are known to be up- or down-regulated in aged TSCs by MTR, it is yet to be determined what signaling pathways are responsible for these changes. Lastly, it is not clear how senescent cells are eliminated or reduced by MTR in aging rat tendons. These parameters will be addressed in future studies.

In conclusion, this is the first study to delve into the effect of exercise on wound healing in aging rats. Our findings show that MTR can promote wound healing in aging rat patellar tendons by inducing several TSC based changes. MTR increased the proliferation of TSCs, expression of stem cell markers and tenocyte related mRNA expression, while decreasing the number of senescent tendon cells present in aging tendons and also suppressing non-tenocyte related gene expression. MTR also increased the organization of the tendon matrix in the wounded tendon. The findings of this study provide new insights and add to the emerging data supporting the role stem cells play in the beneficial effects produced by exercise. Besides, these results also emphasize the importance of pre-exercise; MTR can enhance healing of injured aging tendons by inducing anabolic effects such as increasing TSC proliferation and stemness, which may improve tendon function and quality of tendon repair after an injury. Particularly, these results are relevant to the aging population, who are prone to frequent musculoskeletal injuries and may help devise clinical rehabilitation protocols to treat tendon injuries in aging patients.

## MATERIALS AND METHODS

### Ethics statement

The use of rats for treadmill running, wounding and collecting tendons in this study was approved by the University of Pittsburgh IACUC.

### Treadmill running of aging rats

Aging Sprague-Dawley rats (20 months old and weighing 300-350g) were used for the experiments in this study. For the treadmill running experiment, 24 aging rats were divided into two groups; the cage control group and MTR group with 12 rats in each group. Rats in the MTR group trained for one week by running 15 min/day at the speed of 13 meter/min for 5 days. Then, the rats ran for 30 min/day at the same speed for 5 days/week for 4 weeks based on our previously described protocol [6]. The control rats remained in cages and were allowed unrestricted movement. After MTR, patellar tendons were collected from 3 rats in the control and MTR groups each for morphological and histochemical analyses.

### Aging rat tendon morphology after MTR

Aging rats in the control and MTR groups were anesthetized using isoflurane and the tendon area beneath the skin was exposed. Then, the morphology of rat patellar tendons was observed by visual examination. Images of the tendons were obtained using a camera. A second control group consisting of 6 young rats (3 months) that remained in cages without treadmill running was also used for comparison.

### Histochemical analysis of aging rat tendons after MTR

The effect of MTR on aging rat patellar tendons was determined by histological analysis according to previous descriptions [22]. Briefly, patellar tendons were dissected from 3 rats in each of the control and MTR groups and immersed in frozen section medium (Neg 50; Richard-Allan Scientific; Kalamazoo, MI). Then, they were flash frozen in 2-methylbutane chilled in liquid nitrogen, sectioned (10 μm), dried overnight at room temperature, rinsed in PBS, and fixed in 4% paraformaldehyde for 30 min. Finally, the sections were washed in PBS, stained with H & E and visualized through a microscope.

### Wounding of rat patellar tendons

Immediately after MTR, 6 aging rats each from the MTR and control groups were anaesthetized and wounded in their patellar tendons. These wounds were made by creating a 2-mm window defect on each tendon as described previously [43]. Some of the excised tendons were used to isolate TSCs for *in vitro* culture and some were stored at −80°C for histological analysis. After surgery, the rats were allowed to recover in cages without restricting their movements.

### Evaluating the wounded rat patellar tendons

At two time points (4 and 8 weeks) after the surgery, the rat patellar tendon morphology was visually observed in the control and MTR groups after anesthetizing the animals with isoflurane. Images of the tendons were obtained using a regular camera. The effect of MTR on wound healing in aging rat patellar tendons was also examined by H&E staining after 4 and 8 weeks (3 rats/group/time point), and was performed essentially as described before [22]. Additionally, the wound healing in aging rat patellar tendons after MTR was also examined by staining for β-galactosidase (β-gal), which is a senescent cell marker in aging tissues [44]. The aging marker was stained using a β-gal staining kit (Cell Signaling Technology, Cat. No. 9860, Danvers, MA). Briefly, patellar tendons were flash frozen in 2-methylbutane chilled in liquid nitrogen, sectioned (10 μm) and dried overnight at room temperature. Tissue sections were stained with the reagents provided in the kit following the manufacturer's instructions.

### Isolation of rat TSCs

As previously described [22], TSCs were derived from the patellar tendons excised from 6 rats each in the MTR and control groups during the wounding surgery. First, the patellar tendons were finely chopped and about 100 mg tissue was digested with 3 mg of collagenase type I and 4 mg of dispase in 1 ml of PBS for 1 hr at 37°C. The digested tissue was centrifuged at 3,500 rpm for 15 min and the cell pellet was suspended in Dulbecco's modified Eagle's medium (DMEM; Lonza, Walkersville, MD) with 20% fetal bovine serum (FBS, Atlanta Biologicals Lawrenceville, GA) and 100 U/ml of penicillin and 100 μg/ml of streptomycin (Atlanta Biologicals, Lawrenceville, GA). These cells were cultured in T25 flasks at 37°C and 5% CO_2_ for 10-14 days. Then, TSC colony formation and cell morphology were observed in the MTR and control groups. Cell proliferation in culture was measured using the method described below.

### Measurement of TSC proliferation *in vitro*

TSCs isolated from aging rats in the MTR and control groups were cultured in growth medium (DMEM + 20% FBS) until they reached confluence and the cell number was counted. Then, the following formula was used to calculate the total time taken for the cells to double in number, which is referred to as the population doubling time (PDT): log_2_[Nc/N_0_] where N_0_ is the total number of cells used for seeding, and Nc is the total number of cells at confluence [22].

### Analysis of stem cell marker expression by immunocytochemical staining

Immunocytochemical staining was performed to analyze the expression of the stem cell markers, octamer-binding transcription factor 4 (OCT-4), Nanog, and nucleostemin (NS) in the TSCs from MTR and control groups. First, TSCs (1.5 × 10^4^/well) at passage 1 were seeded in 12-well plates and cultured in DMEM + 20% FBS. After 3 days, cells were fixed in 4% paraformaldehyde/PBS for 30 min at room temperature, washed with 0.1% Triton X-100/PBS for 30 min, rinsed three times in PBS and incubated with specific antibodies for the various stem cell markers including 1:250 times diluted rabbit anti-OCT-4 primary antibody (Abcam, Cambridge, MA, Cat. # ab19857), 1:200 times diluted rabbit anti-Nanog antibody (Abcam, Cambridge, MA, Cat. #ab106465) and 1:350 diluted goat anti-nucleostemin (NS) antibody (Neuromics, Edina, MN, Cat. #GT15050) overnight at 4°C. Excess antibody was removed by washing 3 times in PBS. Then, TSCs were incubated at room temperature for 2 hrs with Cy3-conjugated OCT-4 or Nanog donkey anti-rabbit IgG diluted to 1:500 (Millipore, Billerica, MA, Cat. #AC182C), respectively or with Cy3-conjugated nucleostemin donkey anti-goat IgG (1:500, Millipore, Billerica, MA, Cat. #AC180C). After incubating with the secondary antibodies, TSCs from all immunostaining procedures were washed in PBS and visualized through a fluorescence microscope (Nikon, Eclipse TE2000-U). Image documentation was performed through a digital camera attached to the microscope.

### Semi-quantification of stem cell marker staining

To quantify stem cell marker staining, we used a semi-quantification method as described previously [6]. For each stem cell marker staining, nine images of TSCs were obtained using a camera attached to a Nikon eclipse microscope. The SPOT™ imaging software (Diagnostic Instruments, Inc., Sterling Heights, MI) was used to identify and analyze the positively stained cells. Finally, the percentage of positively-stained cells in the microscopic field was calculated for each of the 9 images/group and the average of all nine images was used as the final percentage of positively stained TSCs in the group.

### Gene expression analysis

qRT-PCR was used to evaluate the expression of stem-cell marker genes (OCT-4, Nanog) [45], tenocyte related genes (Collagen I, Collagen III and tenomodulin) and non-tenocyte related genes (PPAR-γ, Collagen II and Runx-2) in TSCs isolated from the control and MTR rats. First, TSCs at passage 2 were seeded in 6-well plates at a density of 6 × 10^4^/well and cultured for 7 days. Then, the RNeasy Mini Kit (Qiagen, Valencia, CA) was used to isolate total RNA from TSCs based on the manufacturer's instructions. First-strand cDNA was synthesized from about 1 μg total RNA using SuperScript II (Invitrogen, Grand Island, NY) by incubating at 65°C for 5 min, 4°C for 1 min and 42°C for 50 min. About 2 μl of cDNA (total 100 ng RNA) from the first strand reaction was used in a final volume of 25 μl in qRT-PCR performed using the QIAGEN QuantiTect SYBR Green PCR Kit (QIAGEN, Valencia, CA). Temperature cycling conditions included incubation for 5 min at 94°C, followed by 30 to 40 cycles at 94°C for 1 min, 57°C for 40 seconds and 72°C for 50 seconds, and a final incubation at 70°C for 10 min. All reactions were carried out in a Chromo 4 Detector (MJ Research, St. Bruno, Quebec, Canada). The primers used in these reactions were rat-specific and are previously described: OCT-4 and Nanog [45], Collagen III [46], and Collagen I, tenomodulin, PPAR-γ, Collagen II and Runx-2 [5]. All primers were obtained from Invitrogen (Grand Island, NY) and their sequences are shown in Table 1. The housekeeping gene, GAPDH, was used as an internal control to normalize target gene expression [47]. Relative expression levels of target genes were calculated using the formula: 2^−ΔΔCT^, where ΔΔCT = (CT_target_ - CT_GAPDH_)_MTR_ - (CT_target_ - CT_GAPDH_)_control_ and CT represents the threshold cycle of each RNA sample. Each reaction had 3 replicates and all 3 CT values were used to determine the mean expression level. Data are presented as mean ± standard deviation (mean ± SD) for each data set. Expression of each gene in the MTR group was compared to the control group from aging rats.

**Table 1:** RT-PCR primer sequences

Gene	Primer sequence (Forward)	Primer sequence (Reverse)	Reference
Oct 3/4	5′-AGG CAG GAG CAC GAG TGG A-3′	5′-CGA AGC GGC AGA TGG TTG T-3′	45
NANOG	5′-TCT CCT CCG CCT TCC TCT-3′	5′-TTG CCT CTG AAA CCT ATC CTT G-3′	45
Collagen III	5′-GTG CTG CCA TTG CTG GAG TT-3′	5′-CCG GCT GGA AAG AAG TCT GAG-3′	46
Collagen II	5′-ATG ACA ATC TGG CTC CCA ACA CTG C-3′	5′-GAC CGG CCC TAT GTC CAC ACC GAA T-3′	5
PPARg	5′-CGG CGA TCT TGA CAG GAA AG-3′	5′-GCT TCC ACG GAT CGA AAC TG-3′	5
Tenomodulin	5′-GTG GTC CCA CAA GTG AAG GT-3′	5′-GTC TTC CTC GCT TGC TTG TC-3′	5
Runx-2	5′-GCA CCA TGG TGG AGA TCA TC-3′	5′-GTC TGT GCC TTC TTG GTT CC-3′	49
Collagen I	5′-ATC AGC CCA AAC CCC AAG GAG A-3′	5′-CGC AGG AAG GTC AGC TGG ATA G-3′	48
GAPDH	5′-CCA TTC TTC CAC CTT TGA TGC T-3′	5′-AGC TGC AGG AGC AGC AGT GG-3′	48

### Statistical analysis

Data were analyzed by two tailed Student's *t*-test. A difference between the MTR group and control group was considered as statistically significant when *P-*value was less than 0.05.
